# The tight bond between Fanconi anemia and aging

**DOI:** 10.3389/fragi.2026.1752160

**Published:** 2026-02-24

**Authors:** Marco Antonio Mejía-Barrera, Enya Enara Martínez-Torres, Ulises Juárez-Figueroa, Leda Torres, Moisés O. Fiesco-Roa, Benilde García-de-Teresa, Juan Carlos Gomez-Verjan, Jorge Meléndez-Zajgla, Alfredo Rodríguez, Silvia Sánchez, Bertha Molina, Sara Frias

**Affiliations:** 1 Laboratorio de Citogenética, Instituto Nacional de Pediatría (INP), Mexico City, Mexico; 2 Posgrado en Ciencias Biológicas, Universidad Nacional Autónoma de México, Mexico City, Mexico; 3 Instituto Nacional de Geriatría (INGER), Mexico City, Mexico; 4 Laboratorio de Genómica Funcional del Cáncer, Instituto Nacional de Medicina Genómica, Mexico City, Mexico; 5 Departamento de Medicina Genómica y Toxicología Ambiental, Instituto de Investigaciones Biomédicas, Universidad Nacional Autónoma de México, Mexico City, Mexico; 6 Laboratorio de Falla Medular y Carcinogénesis, Instituto Nacional de Pediatría, Mexico City, Mexico

**Keywords:** aging, chromosomal instability, DNA damage, Fanconi anemia, hallmarks of aging, progeroid diseases

## Abstract

Fanconi anemia (FA) is a rare genetic disorder characterized by genomic instability, bone marrow failure, physical abnormalities, and increased cancer susceptibility. Growing evidence suggests that. FA may represent a progeroid syndrome, displaying features of accelerated aging at the cellular and molecular levels. This review examines the cellular and molecular characteristics of FA in the context of the established hallmarks of aging, supporting the hypothesis that FA constitutes a premature aging disorder. The hallmarks of aging, classified as primary, antagonistic, and integrative, are highly interconnected and mutually influential. FA cells exhibit primary hallmarks such as; genomic instability, telomere attrition, epigenetic alterations, and dysregulated autophagy. Antagonistic hallmarks, including cellular senescence, mitochondrial dysfunction, and altered; nutrient sensing, are also evident. Integrative hallmarks, such as stem cell exhaustion, altered; intercellular communication, chronic inflammation, and dysbiosis, arise as downstream consequences of the accumulated primary and antagonistic damage. The presence of these hallmarks, together with the early onset of clinical manifestations such as bone marrow failure, cancer, and premature menopause, strongly supports the notion that FA involves accelerated aging. Although patients with FA lacks the overt physical features typical of other progeroid syndromes, its clinical, cellular, and molecular abnormalities demonstrate a strong association with age-related decline, making FA a valuable model of premature aging. Despite limited experimental evidence directly demonstrating accelerated aging, this review highlights the molecular mechanisms linking FA and aging and identifies understudied areas that warrant further investigation.

## 1 Fanconi anemia

FA is a genetic syndrome with a prevalence in the United States (USA) of 1–5 cases per million live births and a carrier frequency of approximately 1:257–300 (Swift, 1971; Rosenberg et al., 2011). The disease was first described by the Swiss pediatrician Guido Fanconi, who reported a family with three affected siblings presenting with skin hyperpigmentation, growth retardation, hyperreflexia, microcephaly, genital hypoplasia, skeletal alterations of the thumb and radius, and aplastic anemia (Fanconi, 1927). Pathogenic variants (PV) in any of at least 23 FA/BRCA (Fanconi anemia/Breast cancer) pathway genes, nominated *FANCA* through *FANCX* (García-de-Teresa et al., 2020; HUGO Gene Nomenclature Committee, 2023; Kuehl et al., 2025) (see Supplementary Table S1), cause FA. Most of these genes exhibit an autosomal recessive inheritance pattern, except for *FANCB*, which is X-linked, and *FANCR/RAD51*, which follows an autosomal dominant pattern (García-de-Teresa et al., 2020; Kuehl et al., 2025). The FA/BRCA pathway is responsible for detecting and repairing DNA interstrand crosslinks (ICLs), highly deleterious lesions that impede essential cellular processes such as DNA replication and transcription (Semlow and Walter, 2021).

### 1.1 Physical, hematologic, and oncologic FA phenotype

The FA phenotype has been extensively characterized at the clinical level through direct observation of patient features. At the molecular and cellular levels, experimental insights into FA pathophysiology have been obtained *ex vivo* from patient-derived cells, as well as from a variety of experimental models. Primary human samples, including non-immortalized peripheral blood cells and patient-derived fibroblasts harboring pathogenic variants in individual *FANC* genes, enable the direct investigation of cellular states while preserving phenotypic heterogeneity (Auerbach, 2009; Joksic et al., 2012). In parallel, cellular models with targeted disruption of specific *FANC* genes, generated in established cell lines or primary cultures, provide a controlled experimental framework in which wild-type *FANC* genes can be reintroduced to functionally complement the defect, allowing direct comparisons under identical conditions (Nguyen et al., 2023). Finally, genetically engineered murine models carrying complete loss-of-function (knockout, −/−) or partial loss-of-function (knockdown) alleles recapitulate specific cellular, and molecular features of the disease (Bakker et al., 2013; Parmar et al., 2009). Collectively, these complementary models have been instrumental in delineating the molecular mechanisms underlying the physical, hematologic, and oncologic manifestations of the FA phenotype described below.

FA represents the most common hereditary bone marrow failure (BMF) syndrome (Fiesco-Roa et al., 2019; Shimamura and Alter, 2010). The FA phenotype is typically divided into three major categories: 1. Physical abnormalities, 2. Hematologic disorders, 3. Cancer predisposition (García-de-Teresa et al., 2020).

#### 1.1.1 Physical abnormalities

Physical anomalies in FA can affect virtually all organs and systems, resulting in highly variable phenotypic presentations (Hoover et al., 2024). Literature reviews indicate that nearly 80% of patients exhibit at least one physical feature encompassed within the VACTERL-H (Vertebral, Anal, Cardiac, Tracheo-Esophageal, Renal, Limbs, Hydrocephalus) and PHENOS (Pigmentation of the skin, small Head, Eyes, Neurological, Otological, Short stature) acronyms (Fiesco-Roa et al., 2019).

In comprehensively evaluated patients, Altintas et al., described the most frequent features in the Clinic Cohort of the National Cancer Institute in USA: Skin pigmentation abnormalities (82%), Small eyes (82%), Radial ray abnormalities (60%), Otological abnormalities (56%), Microcephaly (50%), Short stature (49%), Renal malformations (37%), Neurostructural abnormalities (37%), Cardiac abnormalities (32%) (Altintas et al., 2023).

Skin pigmentation abnormalities are particularly notable and warrant interpretation in the context of aging. While Ultraviolet (UV) light exposure is the principal exogenous factor contributing to photoaging in the general population (Kim et al., 2022), pigmentary changes in FA are not limited to sun-exposed areas (Ruggiero et al., 2021). This observation suggests that intrinsic mechanisms, such as defective DNA repair FA/BRCA pathway and the accumulation of genomic instability, may also play a role (García-de-Teresa et al., 2020). Furthermore, increasing evidence highlights the role of cellular senescence in skin aging (Kim et al., 2022). FA cells that fail to repair DNA damage may acquire genomic instability that may conduct to senescence (Rosselli et al., 2003). Thus, the pigmentary phenotype observed in FA could reflect molecular processes such as senescence and genome instability that are present in aging.

#### 1.1.2 Hematological disorders

The reduced DNA repair capacity in FA leads to cumulative damage and hematopoietic stem cell depletion, a hallmark feature of the disease (Behrens et al., 2014; Ceccaldi et al., 2012; Domenech et al., 2018). Depletion of hematopoietic stem and progenitor cells (HSPC) in patients with FA starts as early as intrauterine life (Domenech et al., 2018); Consequently, approximately 90% of FA patients develop hematologic abnormalities by age 40 (Kutler et al., 2003), with a median age of bone marrow failure onset of 6.6 years (Altintas et al., 2023). The hematologic spectrum ranges from asymptomatic cytopenia to severe aplastic anemia (AA), myelodysplastic neoplasia (MDS), which has a cumulative incidence of 50% in FA patients aged 50 years and acute myeloid leukemia (AML), with a cumulative incidence of 5% by age 30 (Alter et al., 2018). Macrocytosis is another common feature, present in approximately 90% of patients with FA during early infancy or childhood (Rogers, 2020).

In normal aging, bone marrow cellularity declines from approximately 60% at 20 years of age to about 30% by 70 years, with hematopoiesis becoming progressively restricted to the lower vertebrae, pelvis, and sternum, accompanied by increased marrow adipogenesis (Zhao et al., 2021; Ricci et al., 1990). Aged human pluripotent stem cells (HPSCs) exhibit a bias toward myeloid differentiation, and transcriptional upregulation of genes associated with myeloid hematologic malignancies (Zhao et al., 2021; Pang et al., 2011).

In patients with FA these alterations occur decades earlier and with greater severity, they develop profound bone marrow hypocellularity and pancytopenia, at a median age of ∼7 years, reflecting accelerated HPSC exhaustion; indeed, a reduced marrow cellularity has been detected as early as embryonic development in FA (Ceccaldi et al., 2012; Domenech et al., 2018), and clonal evolution mainly toward myeloid malignancies may emerge before the third decade of life (Alter et al., 2018). Notably, while aging-associated myeloid skewing of hematopoietic stem cells (HSCs) is a well-established feature of normal aging, a comparable intrinsic myeloid bias has only been demonstrated in FA murine models. Young Fancd2^−/−^ Aldh2^−/−^ mice display an HSC transcriptomic signature resembling that of aged wild-type HSCs, characterized by p53 activation and enrichment of myeloid-biased HSCs, providing mechanistic evidence that FA-associated genome instability can drive premature aging-like hematopoietic programs (Wang et al., 2023).

#### 1.1.3 Susceptibility to the development of cancer

Patients with FA have a markedly increased risk of developing neoplasia at an early age. The most frequent cancers are AML and squamous cell carcinomas of the head and neck and the female genital tract, with rates hundreds of times higher than those in the general population (Alter et al., 2018). A study by Alter et al., which included data from the National Cancer Institute Inherited Bone Marrow Failure Syndromes Cohort, revealed the following relative risks among FA patients compared with people without FA: ∼600 for head and neck squamous cell carcinoma (HNSCC), ∼200 for AML, ∼600 for vulvar squamous cell carcinoma (SCC), ∼6,000 for MDS (Alter et al., 2018).

Importantly, the age of onset for these cancers is significantly earlier in patients with FA. In the general population, the median age at cancer diagnosis is 66 years (National Cancer Institute, 2025). Specifically, AML typically arises between 66 and 71 years of age, and cases before age 45 are rare (Nagel et al., 2017). In contrast, FA patients develop AML at a median age of 17 years (Alter et al., 2018). Likewise, non-virus-associated HNSCC occurs at a median age of 66 years in the general population, but at 37 years in individuals with FA (Alter et al., 2018; Johnson et al., 2020). These findings demonstrate that malignancies typically observed after the fifth decade of life appear in FA individuals about 3 decades before, indicating a markedly early onset of cancer and further reinforcing the concept of accelerated aging in this population.

## 2 Aging and progeroid syndromes

There is no single, universally accepted definition of aging, reflecting the diversity of conceptual and methodological approaches to this process (Lemoine, 2020). The World Health Organization (WHO) defines aging as the result of the cumulative impact of a wide range of molecular and cellular damage over time, leading to a progressive decline in physical and mental capacity, an increased risk of disease, and ultimately death (World Health Organization, 2022). Across the heterogeneity of definitions, most definitions converge on two core features: 1) the progressive accumulation of damage over time (Hoeijmakers, 2009; Harman, 2006; Lombard et al., 2005) and 2) the associated functional decline with increased susceptibility to disease and mortality (López-Otín et al., 2013; Kirkwood and Austad, 2000; Kirkwood and Rose, 1991). In contrast, several aspects of aging remain incompletely resolved, including the extent to which aging is reversible. Notably, accumulating evidence indicates that certain genetic, pharmacological, and dietary interventions can delay aging phenotypes, challenging the notion of aging as a strictly unidirectional process (Spadaro et al., 2022; Bernardes de Jesus et al., 2012).

Progeroid syndromes (PSs) comprise a group of rare genetic disorders characterized by an accelerated aging phenotype that closely mimics physiological aging (Hennekam, 2020; Rieckher et al., 2021). The term *progeroid* means “resembling progeria,” derived from the Greek words *pro* and *geras*, meaning “before old age” (Bejaoui et al., 2023). PSs, such as Werner syndrome, Hutchinson–Gilford progeria syndrome, mandibuloacral dysplasia, Nestor–Guillermo progeria syndrome, restrictive dermopathy, ataxia telangiectasia, Bloom syndrome, Cockayne syndrome, Nijmegen breakage syndrome, and Seckel syndrome, exhibit a wide spectrum of pathological manifestations. These clinical features parallel those observed in natural aging and include alopecia, hearing and vision loss, tissue atrophy, skin ulceration, cardiovascular abnormalities, osteoporosis, progressive neurodegeneration, reduced bone marrow cellularity, genomic instability, and an increased susceptibility to cancer. The pathogenic variants underlying these syndromes are functionally linked to genome maintenance and DNA repair, supporting the hypothesis that the accumulation of DNA damage plays a causal role in aging (Hennekam, 2020; Rieckher et al., 2021).

Patients with FA, due to defects in the FA/BRCA DNA repair pathway, exhibit multiple clinical features commonly associated with PSs from early life. These include bone marrow dysfunction, cytopenias, immunodeficiency, chronic inflammation, squamous cell carcinomas, acute myeloid leukemia, endocrine decline, sarcopenia, and osteopenia. In addition, genomic instability and stem cell exhaustion, both recognized hallmarks of aging, are prominent features of FA (Altintas et al., 2023; Kutler et al., 2003; Brosh et al., 2017). The convergence of these phenotypic and molecular characteristics supports the notion that Fanconi anemia may be considered a progeroid syndrome, as it encompasses several features observed across distinct PSs within a single disease entity (Rieckher et al., 2021; Brosh et al., 2017; Velleuer and Carlberg, 2024).

This review seeks to examine the phenotypic and genotypic spectrum of AF within the framework of the hallmarks of aging, to evaluate FA as a premature aging syndrome and to emphasize the need for further research in the less-explored aspects of FA pathophysiology.

## 3 Hallmarks of aging present in Fanconi anemia

The hallmarks of aging were proposed to encompass the fundamental biological processes that drive aging at the cellular, tissue, and organismal levels. According to López-Otín et al., these hallmarks are grouped into three categories, primary, antagonistic, and integrative, based on their hierarchical and functional relationships (López-Otín et al., 2013; López-Otín et al., 2023).

These hallmarks are not isolated mechanisms but rather interconnected processes that collectively contribute to the progressive decline associated with aging. Experimental evidence provides both direct and indirect links between FA and several of these hallmarks, supporting the concept that FA represents a model of premature or accelerated aging (Supplementary Table S2).

### 3.1 Primary hallmarks of aging in Fanconi anemia

**FIGURE 1:**
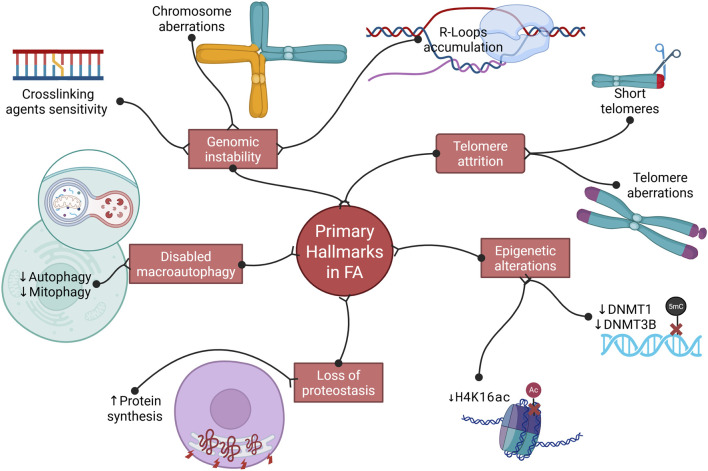
Primary hallmarks of aging related to Fanconi anemia. Fanconi anemia (FA) cells display hypersensitivity to DNA crosslinking agents, resulting in chromosomal aberrations such as radial figures. These cells also accumulate R-loops, highlighting FA as a genomic instability syndrome. Moreover, evidence indicates telomere shortening and structural telomeric abnormalities, consistent with telomere attrition. Epigenetic alterations have also been reported, including reduced expression of DNMT1 and DNMT3β, and decreased acetylation of H4K16. Finally, impaired macroautophagy and increased protein synthesis have been observed in FA HSPCs, leading to defective autophagy and loss of proteostasis. (Created with Biorender).

Primary hallmarks that have unequivocal adverse effects on cellular components. These include genomic instability, telomere attrition, epigenetic alterations, loss of proteostasis, and disabled macroautophagy (López-Otín et al., 2023) (Figure 1).

#### 3.1.1 Genomic instability

Genomic instability is defined as the persistent generation of diverse genetic alterations, ranging from point mutations to large-scale chromosomal rearrangements, and can be classified into chromosomal instability (CIN) and micro- and minisatellite instability (MIN) (Draviam et al., 2004; Aguilera and Gómez-González, 2008). CIN, the most common form of genomic instability in cancer cells, involves a continuous state of mitotic dysfunction and failure in DNA repair pathways, leading to structural and numerical chromosomal abnormalities. MIN results from replication slippage and DNA repair impairment, producing expansions or contractions of repetitive DNA sequences (Bielski and Taylor, 2021).

**FIGURE 2:**
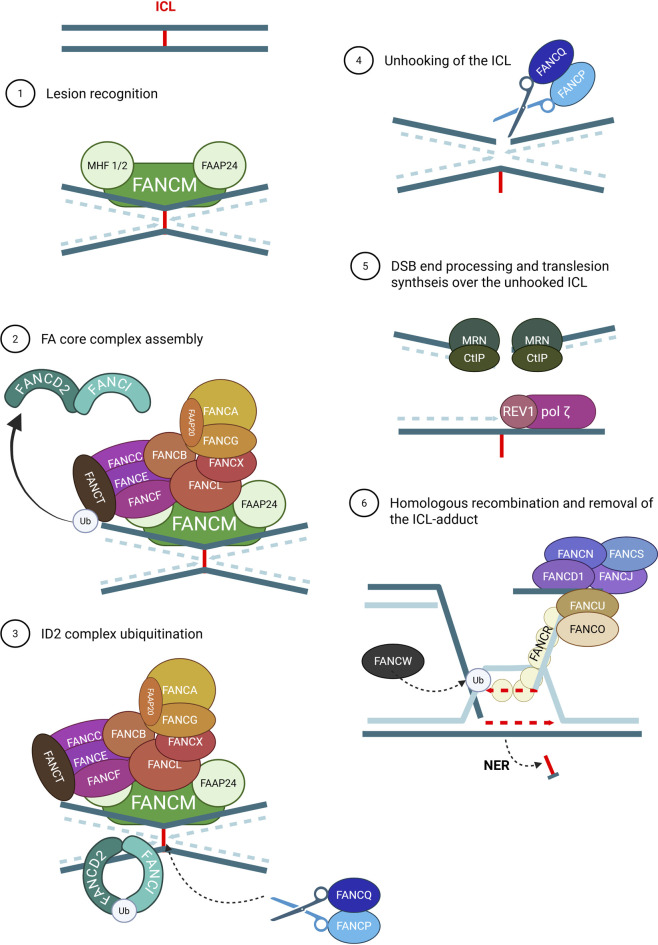
FA/BRCA pathway. FANCM complex recognizes the ICL during replication (1). This triggers the assembly of the FA core complex, involving nine Fanconi proteins and two FA-associated proteins (2), which monoubiquitinates the FANCI-D2 complex (3). This central complex activates exonuclease activity of FANCQ and FANCP, represented as scissors, which unhook the ICL (4). As a result, different lesions must be repaired. On one hand, REV1-polymerase ζ complex performs translesion synthesis on the strand containing the unhooked ICL. At the same time, the DSB is processed by MRN/CtIP (5) and subsequently repaired by homologous recombination. In this process, FANCD1, FANCN, FANCJ, FANCO, and FANCR mediate strand invasion using the sister chromatid as a template to restore the original DNA sequence. Finally, the ICL adduct is removed by nucleotide excision repair (6) (Created with Biorender).

Genomic instability in somatic cells has long been recognized as a causal factor in cell death and degeneration associated with aging, dating back to the 1940s when studies showed that low doses of radiation shorten lifespan and accelerate the accumulation of DNA lesions in late life (Henshaw et al., 1947). Multiple types of DNA alterations contribute to this instability, including chemical damage caused by reactive molecules, such as the formation of interstrand crosslinks (ICLs). Consequently, disruption of ICL repair mechanisms can drive genomic instability (Kottemann and Smogorzewska, 2013). The FA/BRCA pathway plays a central role in ICL repair (Figure 2). It recognizes ICLs through ubiquitin-like with PHD and RING finger domains 1 (UHRF1) and the FANCM-Mph1-associated Histone-Fold (FANCM–MHF1–MHF2) complex. Subsequently, UHRF1 recruits the FANCD2–I complex and the FANCM–MHF1–MHF2 complex to the FA core complex on chromatin. The ubiquitin ligases UBE2T/FANCT and RFWD3/FANCW then transfer ubiquitin to FANCL (a component of the FA core complex), which monoubiquitinates the FANCD2–I heterodimer, facilitating recruitment of the scaffold protein SLX4/FANCP along with the endonucleases MUS81 Structure-Specific Endonuclease Subunit (MUS81), Structure-specific endonuclease subunit SLX1B (SLX1), and XPF/ERCC4/FANCQ, to unhook the ICL (Rodríguez and D’Andrea, 2017). This process generates various DNA repair intermediates that are resolved by distinct pathways: single-stranded DNA fragments are repaired by translesion synthesis; adducts by nucleotide excision repair (NER), and double-strand breaks (DSBs) by homologous recombination (HR) (Rodríguez and D’Andrea, 2017). In normal cells with functional ICL repair, the cell cycle is temporarily arrested to allow DNA repair, and if the damage is extensive or irreparable, apoptosis is triggered (Kottemann and Smogorzewska, 2013; Deans and West, 2011). However, FA cells lacking efficient repair mechanisms may follow two fates: they can undergo apoptosis or survive despite carrying unrepaired DNA damage (Kottemann and Smogorzewska, 2013; Deans and West, 2011; Basu, 2018).

Chromosomal instability in FA cells becomes particularly evident after exposure to DNA crosslinking agents, both endogenous, such as aldehydes, and exogenous, such as diepoxybutane (DEB) or mitomycin C (MMC) (García-de-Teresa et al., 2020; Garaycoechea et al., 2018). FA cells exhibit hypersensitivity to these agents (Auerbach, 2009; Schroeder et al., 1964), and their exposure leads to characteristic chromosomal abnormalities, including chromatid and isochromatid double strand breaks as well as radial figures. These radial formations arise from the joining of breaks between non-sister chromatids, reflecting and maintaining the pronounced chromosomal instability observed in individuals with FA (Hanlon Newell et al., 2008).

An important indicator of genomic instability is the presence of an increase in DNA-RNA hybrid structures known as R-loops. These structures can impede replication fork progression and accumulate when there are defects in DNA repair pathways, such as the FA/BRCA pathway, specifically, FANCD2, FANCA, and BRCA2 play key roles in preventing R-loop-induced DNA damage and replication fork stalling. In human cell lines and primary murine bone marrow cells, deficient in *FANCA* or *FANCD2*, R-loops are markedly enriched compared with FA/BRCA-proficient cells, as detected by DNA-RNA immunoprecipitation-Quantitative Polymerase Chain Reaction (DRIP-qPCR). This finding demonstrates that the FA/BRCA pathway plays a critical role in removing R-loops (García-Rubio et al., 2015). During normal aging, the over-accumulation of R-loops generates DNA damage, disrupting the homeostasis and microenvironment of stem cells (Cinat et al., 2021). R-loop accumulation correlates with reduced lifespan, infertility, epigenetic deregulation, and aberrant expression of transposable elements, all of them observed during aging. Importantly, unresolved R-loops can activate innate immune signaling through the Cyclic GMP–AMP Synthase-Stimulator of Interferon Genes (cGAS–STING) pathway, promoting sterile inflammation. Together, these findings position R-loops as emerging drivers of genomic instability and inflammaging, highlighting their potential role in the molecular mechanisms underlying aging (Zhao et al., 2023).

The bone marrow is a tissue with continuous renewal and constant cell division, each division presenting an opportunity for replicative damage. Individuals with FA are especially susceptible to such damage, which helps explain why they typically develop bone marrow failure at an early age (∼7 years) compared to the general population (∼60 years) (Kutler et al., 2003; Butturini et al., 1994).

#### 3.1.2 Telomere attrition

Because of the unidirectional nature of DNA polymerases and processing events, human telomeres lose approximately 50–200 base pairs with each cell division (Zou et al., 2004). Consequently, telomere shortening occurs after every DNA replication and division cycle. The number of divisions a given cell can undergo before entering senescence is defined as the Hayflick limit (Hayflick and Moorhead, 1961). In humans, telomere length has been shown to correlate with mortality and the incidence of age-related chronic diseases, including Alzheimer’s disease, cardiovascular disease, type 2 diabetes, osteoporosis, and cancer (Cawthon et al., 2003; Gruber et al., 2021; Schneider et al., 2022).

Telomeres in lymphocytes from patients with FA are significantly shorter than those in age-matched controls, as shown by quantitative fluorescence *in situ* hybridization (Q-FISH) analysis of 16 FA patients, revealing a marked reduction in telomere length (Hanson et al., 2001). Similarly, analyses of lymphocytes and fibroblasts from patients with *FANCD2* mutations identified multiple telomeric abnormalities, such as premature telomere shortening, increased telomeric recombination, and aberrant telomeric structures (Joksic et al., 2012). More recently, telomere length was quantified by real-time quantitative polymerase chain reaction (qPCR) in individuals carrying *FANCA, FANCG*, and *FANCL* mutations. All three FA groups exhibited significant telomere shortening compared with controls, with individuals from the *FANCL* group showing the most pronounced reduction (Shah et al., 2021).

Other studies have indirectly implicated FANC proteins in telomere maintenance. Although no changes in basal telomere length were observed in a *Fancc* knockout mouse model, increased telomere attrition became evident when bone marrow cells were subjected to high proliferative stress through serial transplantation. These findings suggest that *Fancc* deficiency accelerates telomere shortening during rapid proliferation of hematopoietic cells (Rhee et al., 2010).

An alternative pathway for telomere maintenance is the alternative lengthening of telomeres (ALT) mechanism, in which several FANC proteins play key roles. FANCD2 colocalizes with the telomeric protein Telomeric Repeat Binding Factor 1 (TRF1) exclusively in ALT-positive cells, and depletion of FANCA or FANCD2 results in telomere loss and reduced telomere sister chromatid exchange (Fan et al., 2009). Similarly, loss of FANCM in ALT cells induces severe telomeric replication stress and heightened ALT activity, reflecting the role of specific FANCM domains in suppressing ALT (Lu et al., 2019; Silva et al., 2019). Furthermore, FANCJ unwinds guanine quadruplex (G4) DNA structures *in vitro* (London et al., 2008), and its depletion leads to impaired proliferation, increased apoptosis, and accumulation of DNA damage (Wu et al., 2008). Collectively, these findings highlight the potential contribution of FANC proteins to telomere homeostasis and suggest that their role in Fanconi anemia warrants further investigation.

#### 3.1.3 Epigenetic alterations

Epigenetic alterations contribute to aging by modulating the expression of genes involved in diverse pathways, including cellular stress response, senescence, mitochondrial dysfunction, and telomere attrition. These alterations can impact multiple mechanisms that govern gene expression through changes in chromatin structure, such as DNA methylation patterns, post-translational modifications of histones, chromatin remodeling, and dysregulation of non-coding RNAs (Jusic et al., 2022; Oh and Petronis, 2021; Seale et al., 2022; Swer and Sharma, 2021). Aging-associated diseases, including cardiovascular disorders, neurodegenerative conditions, and cancer, are all linked to changes in epigenetic regulation (la Torre et al., 2023).

The Information Theory of Aging (ITOA) posits that biological information is stored in two main forms: the DNA nucleotide sequence and the epigenome. This theory explains why individuals with unique genomes can exhibit similar aging phenotypes. Unlike the somatic mutation theory of aging, the ITOA emphasizes the epigenome’s role in regulating multiple cellular processes, including DNA repair (Lu et al., 2023). According to the ITOA, cellular responses to damage originate from chromatin alterations and epigenetic dysregulation, which sensitize cells to DNA damage (Yang et al., 2023).

Despite its relevance, few studies have addressed epigenetic regulation in FA. Peripheral blood mononuclear cells from FA patients display an epigenetic profile distinct from healthy individuals. Consistent with the ITOA, these cells exhibited reduced expression of the DNA methyltransferases DNA Methyltransferase 1 (DNMT1) and DNA Methyltransferase 3β (DNMT3β) (Belo et al., 2015). Such alterations in methylation machinery may underlie the FA-specific epigenetic signature recently identified, which has potential diagnostic utility (Pagliara et al., 2023).

Other levels of epigenetic regulation are also affected in FA. In *FANCC*-deficient fibroblasts, a significant decrease in H4K16 acetylation was observed (Renaud et al., 2016). This mark, catalyzed by the Tat-interacting protein, 60 kDa (TIP60) enzyme, normally inhibits recruitment of the non-homologous end-joining (cNHEJ) protein p53-binding protein 1 (53BP1) to its docking site, H4K20Me2, thereby preventing repair *via* the error-prone cNHEJ pathway. The reduction in H4K16 acetylation, together with the accumulation of 53BP1, Replication Timing Regulatory Factor 1 (RIF1), and Receptor-Associated Protein 80 (RAP80) at damaged chromatin, suggests that double-strand breaks in FA-deficient cells are preferentially repaired through error-prone pathways (Renaud et al., 2016).

Similarly, hypoacetylation of histones has been reported in FANCA-deficient cells, particularly at replication forks. While these acetylation changes were not immediately apparent after acute DNA damage, chemically modifying histone acetylation in these cells induced DNA damage, indicating a critical role for proper acetylation in maintaining genomic stability (García-de-Teresa et al., 2024).

Gene expression studies in FA have also highlighted the influence of epigenetic regulation. The active form of vitamin D affects the transcription of over 1,000 genes, with *FANCE* among its potential targets. Vitamin D receptor (VDR) binding enhances chromatin accessibility and increases the active chromatin mark H3K27ac at the *FANCE* locus, suggesting that vitamin D insufficiency could modulate the progression of genetic disorders such as FA (Velleuer and Carlberg, 2020).

Finally, dysregulation of non-coding RNAs has been observed in FA. In FANCP/SLX4-deficient cells, the Long interspersed nuclear element-1 (LINE-1) retrotransposable elements, typically silenced by 5′-UTR methylation, show increased retrotransposition. This loss of epigenetic silencing is accompanied by elevated production of proinflammatory cytokines, implicating these mobile elements in cancer susceptibility in patients with FA (Brégnard et al., 2016).

#### 3.1.4 Loss of proteostasis

Loss of proteostasis, or altered protein homeostasis, is a hallmark of physiological aging, characterized by the accumulation of misfolded proteins or proteins modified by glycosylation, oxidation, or ubiquitination. This process has been implicated in natural aging as well as in neurodegenerative diseases such as Alzheimer’s and Parkinson’s disease (Hipp et al., 2019). To date, only one study has reported alterations in protein homeostasis in FA. In this study, hematopoietic stem and progenitor cells (HSPCs) from *Fancd2^−/−* fetal mouse liver exhibited increased protein synthesis and accumulation of misfolded proteins, which induced endoplasmic reticulum stress, restricted HSPC expansion during gestation and inflammaging (Kovuru et al., 2024); all these processes observed in natural aging are recapitulated by FA in early stages of development, however, they remain limited to murine models. Further research is required to establish whether this phenomenon is a consistent feature in patients with FA.

#### 3.1.5 Disabled macroautophagy

Macroautophagy refers to a set of cellular processes in which cytoplasmic components are sequestered within double-membrane vesicles that subsequently fuse with lysosomes or vacuoles to degrade their contents (Yamamoto et al., 2023). Importantly, autophagy is not limited to protein quality control but also regulates other macromolecules, damaged organelles, such as mitochondria (mitophagy), lysosomes (lysophagy), and invading pathogens (xenophagy) (Levine and Kroemer, 2019).

In mouse embryonic fibroblasts, deletion of *Fancc* reduces the colocalization of the Sindbis virus capsid protein with autophagosomes. Consistently, *Fancc*-deficient mice exhibit increased susceptibility to Sindbis virus and herpes simplex virus type 1 (HSV-1) infection, indicating that FANCC functions as a key adaptor in antiviral defense in the central nervous system through autophagy-dependent pathways (Sumpter et al., 2016). In addition, accumulation of damaged mitochondria in brain and heart cells of *Fancc*-deficient mice implicates FANCC in mitophagy. Mechanistically, FANCC interacts with the ubiquitin ligase parkin, which also associates biochemically with FANCA, and several FA proteins, including FANCA, FANCL, FANCF, FANCD2, FANCS, and FANCD1, have been shown to contribute to parkin-mediated mitophagy by siRNA studies, (Sumpter et al., 2016). This role is further supported by viral infection models, as Zika virus induces autophagy in mouse and human neural stem cells while deregulating key selective autophagy genes, including *FANCC*. Loss of *FANCC* activity compromises selective autophagy and mitophagy, thereby facilitating Zika virus replication. These observations underscore the importance of FA proteins in antiviral defense and macroautophagy (Tiwari et al., 2020).

In peripheral blood mononuclear cells from patients with FA, it has been observed a decreased expression of autophagy-related genes, including Autophagy Related 3 (*ATG3*)*,* Autophagy Related 4B Cysteine Peptidase (*ATG4B*)*,* Autophagy Related 7 (*ATG7*)*,* Autophagy Related 12 (*ATG12*)*,* Autophagy Related 16 (*ATG16*)*,* Microtubule-Associated Protein 1 Light Chain 3 Beta (*MAP1LC3B*), and Beclin 1 (*BECN1*), suggesting impaired autophagy. This was accompanied by overactivation of the Notch signaling pathway, which is involved in intercellular communication (Zipporah et al., 2020).

Finally, in HeLa cells *FANCL*-deficient using clustered regularly interspaced short palindromic repeats/CRISPR-associated protein 9 (CRISPR/Cas9), parkin is overexpressed and sensitivity to mitochondrial stress is increased. Restoration of wild-type *FANCL* or expression of the FANCLC307A mutant, which lacks ubiquitin ligase activity, corrected these phenotypes, indicating that FANCL protects against mitochondrial stress and supports parkin-mediated mitophagy, independently of its ligase function (Beesetti et al., 2022).

Collectively, these findings highlight a critical role for FANC proteins in autophagy and mitophagy, linking defective autophagic processes to cellular stress responses in FA. The evidence related to the primary hallmarks of aging is summarized in Figure 1. These hallmarks serve as a common denominator in aging and directly influence the subsequent antagonistic and integrative hallmarks, as discussed below.

### 3.2 Antagonistic hallmarks of aging in Fanconi anemia

**FIGURE 3:**
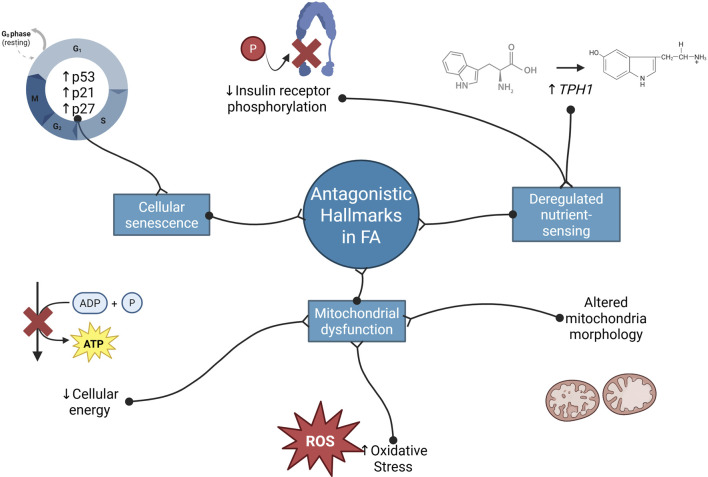
Antagonistic hallmarks of aging related to Fanconi anemia. FA cells exhibit main features of senescent cells such p53, p21, and p27 overexpression. Furthermore, these cells present a reduction of ATP production, increase oxidative damage and mitochondrial abnormalities, suggesting mitochondrial dysfunction. Finally, FA cells evidence deregulation in nutrient sensing such as dysfunction of the insulin signaling pathway and overexpression of *TPH1* (Created with Biorender).

Antagonistic hallmarks counteract primary hallmarks, often acting as protective responses to cellular, tissue, or organ damage. However, their persistent or excessive activity can be deleterious, contributing to aging and disease. These hallmarks include cellular senescence, mitochondrial dysfunction, and deregulated nutrient sensing (López-Otín et al., 2013; López-Otín et al., 2023). The evidence for these processes in FA is summarized in Figure 3.

#### 3.2.1 Cellular senescence

Cellular senescence is characterized by irreversible cell cycle arrest and resistance to apoptosis (Huang et al., 2022). It is triggered by stress or genomic damage, including DNA breaks and telomere shortening, and serves as a tumor-suppressive mechanism (Harley et al., 2024; Helbling-Leclerc et al., 2021; Toropov et al., 2023). Senescent cells accumulate during natural aging and age-related diseases and contribute to cancer development (Calcinotto et al., 2019); it can propagate through tissues *via* paracrine signaling mediated by Senescence-associated secretory phenotype (SASP) components such as C-C Motif Chemokine Ligand 2 (CCL2), Transforming Growth Factor-beta (TGF-β), Interleukin-1 (IL-1), Interleukin-6 (IL-6), and Interleukin-8 (IL-8) (Birch and Gil, 2020; Gorgoulis et al., 2019). Cellular senescence is a context-dependent program, important for normal development, regeneration, and tumor suppression when transient, whereas SASP associated with chronic senescence, as seen with aging, is considered a detrimental process (Moiseeva et al., 2023; Lavarti et al., 2025).

Evidence from cellular and murine models indicates that disruption of the FA/BRCA pathway is sufficient to induce senescence. In human melanoma cell lines, silencing of *FANCA* or *FANCD2* results in increased cell size and granularity (features that are commonly associated with cellular senescence) together with elevated levels of p53, p21, and p27, increased senescence-associated β-galactosidase (SA-βGal) activity, accumulation of senescence-associated heterochromatin foci (SAHF), and heightened reactive oxygen species (ROS) (Bourseguin et al., 2016; Helbling-Leclerc et al., 2019); consistently, FA mesenchymal stem cells display aberrant cytokine profiles, including SASP (Han et al., 2022; Haga et al., 2023; Korthof et al., 2013; Epanchintsev et al., 2015). Persistent DNA damage and ROS accumulation are therefore proposed to sustain a chronic senescent state in FA cells, reinforcing a loop of inflammation and cellular dysfunction (Helbling-Leclerc et al., 2021).

In FA human embryo cells, chronic replication stress and defective interstrand crosslink repair promote unresolved DNA damage and sustained activation of the p53-p21 axis, leading to early-onset senescence at markedly younger biological ages (Ceccaldi et al., 2012; Nalepa and Clapp, 2018). Persistent senescence in this context is predicted to establish a SASP, thereby driving sterile inflammation, tissue dysfunction, and hematopoietic stem cell attrition. Thus, premature and unresolved senescence in FA closely mirrors pathological aging-associated senescence and likely contributes to the accelerated aging phenotypes characteristic of the disease.

#### 3.2.2 Mitochondrial dysfunction

Mitochondrial function declines with age, leading to increased ROS production, inflammasome activation, and Mitochondrial DNA (mtDNA) release, which can trigger inflammation and compromise mitochondrial integrity (Guo and Chiang, 2022). Since the discovery of mitochondrial dysfunction in FANCG-deficient cells (Mukhopadhyay et al., 2006), evidence has accumulated for an essential role of FANC proteins in mitochondrial homeostasis (Ravera et al., 2013; Cappelli et al., 2017; Kumari et al., 2014).

Mitophagy is critical for removing damaged mitochondria, reducing ROS, and preventing cell death. In FA, mitophagy is impaired, leading to oxidative damage and disease progression (Sumpter et al., 2016). FA cells exhibit a pro-oxidant state, partly due to defective repair of ROS-induced DNA lesions, as evidenced by high levels of 8-oxo-deoxyguanosine in patient DNA (Degan et al., 1995; Myers et al., 2011).

Mitochondrial dysfunction has been documented in FANCD2^−/−^ fibroblasts and FANCA^−/−^ lymphoblasts, with increased ROS, reduced mitochondrial membrane potential, decreased oxygen consumption, altered morphology, and low Adenosine Triphosphate (ATP) levels. These defects result from inactivation of F1F0ATPase and cytochrome C oxidase, impairing cellular energy production (Kumari et al., 2014). FANCD2 also participates in mito-nuclear communication, regulating the mitochondrial unfolded protein response (mt-UPR) and transcription at common fragile sites *via* the FANCD2/FANCI- Ubiquitin-Like Protein 5 (UBL5) axis to prevent dysfunction (Fernandes et al., 2021).

Interestingly, certain FANCA pathogenic variants are associated with milder mitochondrial phenotypes, showing intermediate ATP/Adenosine Monophosphate (AMP) ratios between wild-type and null cells, suggesting hypomorphic effects on mitochondrial activity (Bottega et al., 2018). The severity of mitochondrial dysfunction in FA has led to the proposal that FA is, in part, a mitochondrial disease, with mitochondrial defects contributing to diabetes, impaired glucose tolerance, and other metabolic abnormalities (Pagano et al., 2021; Pagano et al., 2020; Sing et al., 2022).

#### 3.2.3 Deregulated nutrient sensing

The nutrient-sensing cellular network includes extracellular ligands, receptors, and signaling cascades. Nutrient-sensing pathways detect extracellular nutrients and stress, balancing anabolic and catabolic processes. Aging disrupts this balance, impairing cellular responses to nutrition and stress (López-Otín et al., 2023). Key nutrient-sensing regulators such as AMP-activated protein kinase (AMPK), sirtuins (SIRTs), insulin, insulin-like growth factor 1 (IGF1), and mechanistic target of rapamycin (mTOR) signaling are central to aging biology, as they coordinate cellular energy status with metabolic homeostasis, stress responses, and longevity. Nutrient-sensing pathways are recognized as fundamental regulators of aging.

In FA, many individuals display disrupted appetite and eating behavior (“picky eating”), which may be linked to alterations in nutrient-sensing hormonal responses, including those mediated by ghrelin, which has been found markedly reduced in patients with FA (Velleuer and Carlberg, 2024; Vicente-Muñoz et al., 2025). This phenotype is accompanied by endocrine dysfunction, insulin resistance, and early-onset type 2 diabetes, suggesting intrinsic disturbances in nutrient-sensing pathways that parallel aging-related metabolic processes. Several studies in patients with FA support this notion, reporting high prevalences of metabolic abnormalities, including impaired glucose regulation and insulin resistance, ranging from 30% to 80% (Hoover et al., 2024; Altintas et al., 2023; Vicente-Muñoz et al., 2025; Petryk et al., 2015). Notably, these values are comparable to those observed in adults aged ≥65 years in the non-FA population (Zhong et al., 2021; Simarro et al., 2011) and are significantly higher than those reported in age-matched non-FA populations (Rogero Blanco et al., 2012; Nolan et al., 2017; Luk et al., 2025).

Dynamic metabolic studies have demonstrated in patients with FA, a great alteration in the substrate utilization, characterized by insulin resistance, sustained hyperglycemia, and a failure to increase postprandial energy expenditure, indicating defective insulin/IGF1 signaling and reduced metabolic flexibility (Vicente-Muñoz et al., 2025), showing a preferential shift toward lipid utilization and enhanced ketogenesis that suggests compensatory activation of energy-conserving pathways typically associated with AMPK and SIRT signaling pathways. This imbalance between anabolic (insulin/IGF1–mTOR) and catabolic (AMPK–SIRT) nutrient-sensing pathways mirrors age-related metabolic remodeling but occurs at markedly younger ages and in the absence of classical obesity. (Velleuer and Carlberg, 2024; Vicente-Muñoz et al., 2025). In line with this, amino acid metabolism in individuals with Fanconi anemia (FA) is altered at both the cellular and systemic levels. FA cells exhibit dysregulation of amino acid sensing and utilization, particularly involving the branched-chain amino acids isoleucine and valine, the aromatic amino acids phenylalanine and tyrosine, glycine (Vicente-Muñoz et al., 2025). In addition, tryptophan metabolism is disrupted, favoring its conversion to serotonin, a metabolite implicated in oncogenesis (Bartlett et al., 2021); these alterations support an insulin-resistant and metabolically abnormal phenotype (Vicente-Muñoz et al., 2025; Li et al., 2012).

Collectively, these data raise the hypothesis that FA represents a model of accelerated yet metabolically atypical aging, influenced by nutrigenomic and lifestyle-related factors that induce cellular disturbances in signal transduction pathways. These disturbances may in turn, affect the epigenome, particularly the DNA methylome, thereby influencing patients’ biological age. Probing the existence of such epigenetic changes may enable the monitoring of modifications in the environment and food intake in the patients with FA, and the identification of druggable targets to delay or even reverse age-related features and diseases. (Velleuer and Carlberg, 2024).

### 3.3 Integrative hallmarks

**FIGURE 4:**
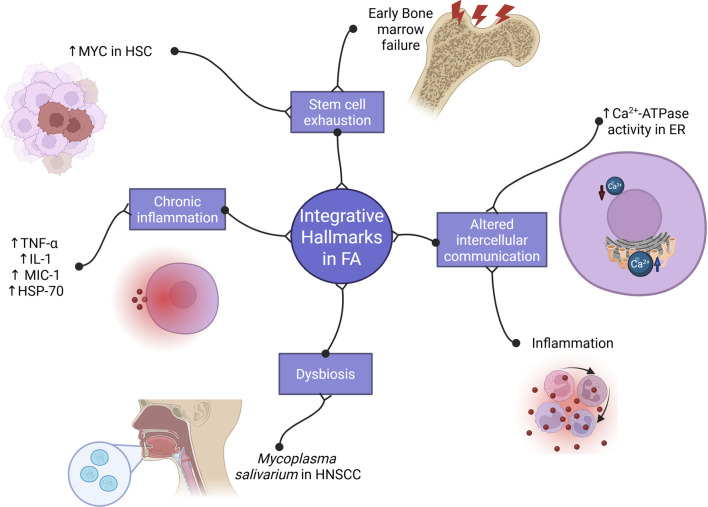
Integrative hallmarks of aging related to Fanconi anemia. FA cells exhibit molecular features of chronic inflammation as well as increase of TNF-α, IL-1, MIC-1 and HSP-70. Furthermore, this chronic inflammation with the altered Ca^2+^ ATPase altogether suggests malfunctioning of intercellular communication. Otherwise, patients with FA presents with early bone marrow failure that shows stem cell exhaustion. Finally, there is some evidence of *Mycoplasma salivarium* abundancy that might be signal of dysbiosis (Created with Biorender).

Integrative hallmarks emerge when accumulated damage from primary and antagonistic hallmarks overwhelms cellular compensatory mechanisms, impairing tissue function and homeostasis (López-Otín et al., 2023). These hallmarks include stem cell exhaustion, altered intercellular communication, chronic inflammation, and dysbiosis. Evidence for these processes in FA is summarized in Figure 4.

#### 3.3.1 Stem cell exhaustion

Aging is characterized by a progressive decline in the self-renewal capacity of tissues (López-Otín et al., 2023). HSPCs in the bone marrow gradually lose regenerative potential and exhibit skewed differentiation toward the myeloid lineage (Shevyrev et al., 2023). This decline compromises tissue homeostasis and is influenced by other hallmarks, including genomic damage, senescence, autophagy defects, and epigenetic alterations (Childs et al., 2015; Ho et al., 2017; Walter et al., 2015; Adelman et al., 2019).

In FA, HSPC exhaustion underlies BMF, which affects up to 90% of patients by age 50 (Kutler et al., 2003; Alter et al., 2018). BMF may manifest as isolated cytopenia or progress to AA, MDS, or AML (Altintas et al., 2023). Mesenchymal stromal cells (MSCs) from patients with FA show premature senescence, spontaneous chromosomal breaks, lower fibroblast-colony-forming unit (CFU-F) counts, and reduced proliferative capacity compared to healthy donors (Mantelli et al., 2015).

Mouse models reinforce these observations. *Fancc/Fancg* double knockout (DKO) MSCs exhibit reduced survival, lower CFU-F numbers, bone marrow hypoplasia, and skewed differentiation toward adipocytes during osteoblast differentiation. This shift disrupts the normal niche activity to maintain hematopoiesis as adipocytes negatively regulate hematopoietic activity and represent an important source of TNF-α which induce apoptosis in the FA hematopoietic cells. Collectively, these features contributes to hypoplasia and BMF (Zhou et al., 2017). MSCs from FA patients, including a single *FANCG*-mutant sample, display increased adipogenic differentiation, elevated mitochondrial ROS, and transcriptomic signatures of senescence, consistent with accelerated aging. (Haga et al., 2023; Zhou et al., 2017).

Endogenous and exogenous genotoxic stress is elevated in FA HSPCs. Reactive aldehydes contribute to DNA damage, as indicated by γH2AX accumulation (Langevin et al., 2011), indeed, FA patients with Aldehyde Dehydrogenase 2 (ALDH2) deficiency, a critical enzyme for acetaldehyde detoxification, develop BMF within the first 7 months of life, highlighting the importance of aldehyde clearance in hematopoietic homeostasis (Hira et al., 2013). Moreover, MYC proto-oncogene overexpression in FA HSPCs occurs in the context of chronic activation of the TGF-β and p53 signaling axes, which promotes transcriptional reprogramming promoting the downregulation of cell adhesion-related genes, resulting in impaired adhesion to the bone marrow niche, and increased susceptibility to HSPC detachment, exhaustion, and bone marrow failure (Rodríguez et al., 2021a; Rodríguez et al., 2022; Rodríguez et al., 2021b; Zhang et al., 2016).

#### 3.3.2 Alteration of intercellular communication

Aging disrupts signaling pathways, resulting in impaired intercellular communication that affects systemic tissue function (López-Otín et al., 2013). In FA, altered intra and intercellular communication is a well-documented feature and contributes to bone marrow failure, and cancer predisposition. It is evident that the homeostasis of calcium, an indispensable element for cell signal transmission, is impaired in FA-A fibroblasts, with threefold lower basal intracellular Ca^2+^ compared to wild-type or *FANCA*-corrected cells, and Ca^2+^ is concentrated in the endoplasmic reticulum due to heightened Ca^2+^-ATPase activity (Usai et al., 2015; Luan and Wang, 2021).

HSPC in FA display defective communication with the bone marrow niche, this due to dysregulated adhesion molecules and chemokine signaling CXCL12–CXCR4 axis that mediates cell-cell communication regulating homing, survival and retention of HSPCs within the bone marrow niche, leading to impaired niche retention and stem cell exhaustion (Rodríguez et al., 2021a). FA cells are also hypersensitive to TGF-β signaling, resulting in aberrant paracrine signaling, enhanced p53 activation, and suppression of HSPC proliferation and self-renewal (Rodríguez et al., 2021a; Rodríguez et al., 2022; Rodríguez et al., 2021b). In addition, chronic DNA damage and replication stress promote inflammatory signaling and SASP-like cytokine secretion, contributing to the propagation of senescence-associated paracrine signaling and to inflammaging (Helbling-Leclerc et al., 2021). This represents an altered form of intercellular communication characterized by the sustained production of proinflammatory cytokines, leading to disrupted tissue homeostasis (López-Otín et al., 2013).

Together, these findings indicate that FA is characterized not only by intrinsic DNA repair defects but also by profound alterations in intercellular communication at the tissue level.

#### 3.3.3 Chronic inflammation

Inflammaging refers to the progressive accumulation of inflammatory mediators with age, including IL-6, TNF-α, N-terminal pro-B-type natriuretic peptide (NT-proBNP), Cystatin C, and cholinesterase, which are associated with increased mortality and immune decline (Hirata et al., 2020; Milan-Mattos et al., 2019; Mogilenko et al., 2021). Chronic inflammation arises from multiple hallmarks, including genomic instability, epigenetic dysregulation, proteostasis loss, autophagy defects, telomere attrition, mitochondrial dysfunction, cellular senescence, stem cell depletion, and altered intercellular communication (López-Otín et al., 2023).

In FA, bone marrow dysfunction is exacerbated by immune-regulatory cytokines such as Interferon-gamma (IFN-γ) (Li et al., 2004; Si et al., 2006). This suggests that increased concentrations of cytokines are involved in the evolution of BMF in individuals with FA; however, the precise mechanism is unknown. DNA damage and chronic stress contribute to inflammation: FANCC-deficient cells overexpress inflammatory mediators, including Interleukin-1 Receptor Antagonist (IL-1RA), Macrophage Inhibitory Cytokine 1 (MIC-1), Heat Shock Protein 70 (HSP-70), and accumulate TNF-α to levels 5–8 times higher than healthy controls, suggesting that in addition to its function in DNA repair, FANCC might play a role in inflammatory response, which could influence the inflammatory phenotypes observed in FA (Za et al., 2004; Briot et al., 2008). On the other hand, FANCD2 and FANCC deficiency enhances TNF-α induced Nuclear Factor kappa-light-chain-enhancer of activated B cells (NF-Κb) activation, sustaining inflammatory activity (Matsushita et al., 2011). Additionally, overexpression of IL-1β in FA mononuclear cells can be rescued by FANCA gene complementation, further linking FA gene deficiency to inflammatory dysregulation (Ibáñez et al., 2009).

Collectively, FA cells display a proinflammatory phenotype that reflects the cumulative effect of deficiencies in DNA repair, mitochondrial function, senescence control, and other cellular mechanisms.

#### 3.3.4 Dysbiosis

Dysbiosis is characterized by disrupted host-microbiota communication, including reduced beneficial organisms, overgrowth of harmful species, or decreased microbial diversity. Notably, these categories are not mutually exclusive and often coexist (DeGruttola et al., 2016). In aging, dysbiosis is associated with reproducible alterations in gut microbiota composition and function, and is characterized by reduced microbial diversity, depletion of short-chain fatty acid–producing commensals, and enrichment of pro-inflammatory pathobionts (O’Toole and Jeffery, 2015). Data regarding this hallmark is limited in FA, however there is evidence that patients with FA carry a local microbial imbalance within the oral niche, in terms of the elevated presence of *Streptococcus, Neisseria* and *Haemophilus*, specifically in patients presenting with oral lesions and severe mucositis (Furquim et al., 2017). Additionally, *Mycoplasma salivarium* was abundant on the surface of a squamous cell carcinoma in an FA patient, compared to benign lesions and healthy controls, implicating dysbiosis in cancer progression (Henrich et al., 2014). Altered microbiota composition has also been linked to head and neck squamous cell carcinoma, although not performed in FA patients, this is the most common solid tumor in patients with FA (Alter et al., 2018; Ting et al., 2023).

While underexplored, dysbiosis may contribute to aging phenotypes and cancer susceptibility in FA and warrants further investigation.

## 4 Interplay between primary, antagonistic and integrative hallmarks in Fanconi anemia

**FIGURE 5:**
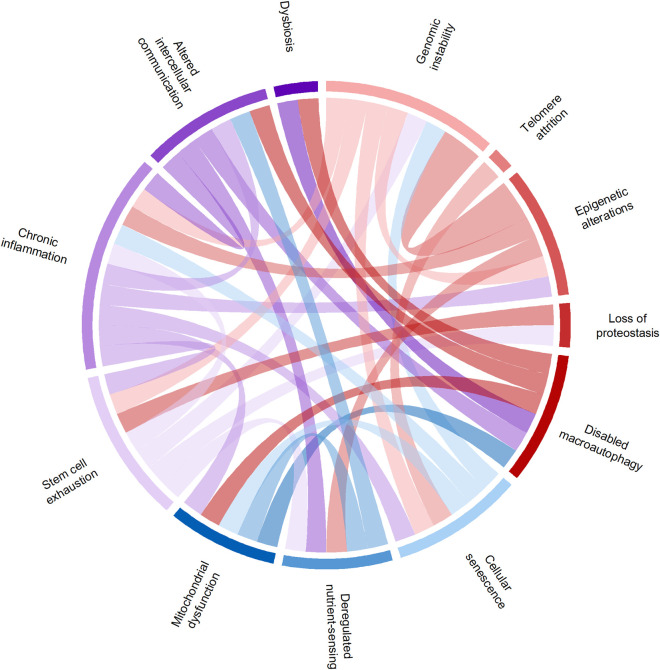
Circus plot of interactions between aging hallmarks. Each ribbon representing an interaction. The red color represents the primary hallmarks, the blue color the antagonistic hallmarks and the purple the integrative hallmarks.

Fanconi anemia is a genetic syndrome with a median survival of 39 years (Alter et al., 2018), characterized by multiple cellular and molecular features that overlap with aging. At the molecular level, the hallmarks of aging are highly interconnected, creating a feedback network that links primary sources of damage to downstream consequences (Figure 5).

### 4.1 Connections from primary hallmarks

Genomic instability, a central primary hallmark, drives the accumulation of DNA damage that can activate cellular senescence (Helbling-Leclerc et al., 2021), and impair stem cell function, particularly evident in HSPCs (Cinat et al., 2021; Schuma et al., 2021). Telomere shortening further contributes to senescence and genomic instability (Harley et al., 2024).

R-loop accumulation, a byproduct of defective DNA repair, represents another intersection of hallmarks. While R-loops are associated with genomic instability and cancer, they also influence chromatin condensation and epigenetic gene silencing, linking DNA damage to epigenetic regulation (Kim and Wang, 2021). Similarly, LINE-1 retrotransposition contributes to chronic inflammation (Brégnard et al., 2016), and reduced DNMT3B expression correlates with chromosomal instability (Belo et al., 2015). Decreased H4K16 acetylation activates error-prone repair, promoting chromosomal aberrations, while experimental histone hyperacetylation paradoxically induces DNA damage, likely by disrupting protective chromatin structures against R-loop accumulation (García-de-Teresa et al., 2020; García-de-Teresa et al., 2024). This evidence suggests a strong relationship between DNA damage and epigenetics.

Altered autophagy mechanisms also integrate multiple hallmarks. Defective virophagy increases susceptibility to viral infections, exemplified by FA neuroinflammatory syndrome (FANS), where polyomavirus-infected microglia are observed (Bartlett et al., 2024), suggesting a connection with dysbiosis. Impaired autophagy coupled with upregulated Notch signaling alters intercellular communication (Zipporah et al., 2020; Bigas and Espinosa, 2016), while deficient mitophagy prevents the clearance of dysfunctional mitochondria, promoting oxidative stress (Fernandes et al., 2021).

Although data are limited, loss of proteostasis in FA has been linked to HSPC restriction during embryogenesis, illustrating another connection between primary damage and integrative outcomes (Kovuru et al., 2024).

### 4.2 Connections to antagonistic and integrative hallmarks

Primary hallmarks feed directly into antagonistic and integrative hallmarks. Mitochondrial dysfunction generates elevated ROS, which drives oxidative stress, induces senescence (Helbling-Leclerc et al., 2021; Bourseguin et al., 2016), and impairs insulin signaling, contributing to metabolic dysregulation (Li et al., 2012). SASP factors secreted by senescent cells can interact with adjacent cells mediate altered intercellular communication and propagate inflammation (Birch and Gil, 2020). Increased serotonin levels in FA may also affect metabolic and signaling pathways beyond neurotransmission (Bartlett et al., 2021).

Ultimately, Endogenous aldehyde accumulation, a metabolic byproduct, exacerbates early HSPC exhaustion, linking metabolic dysregulation to stem cell depletion. Chronic inflammation further reinforces this process: proinflammatory FA cells produce cytokines that either directly promote HSPC attrition or create a microenvironment that drives elevated MYC expression, amplifying replicative and genotoxic stress (Rodríguez et al., 2021a).

## 5 Integrative perspective

The cumulative effect of primary and antagonistic hallmarks manifests as integrative hallmarks, including stem cell depletion, chronic inflammation, altered intercellular communication, and potentially dysbiosis. Together, these processes compromise tissue and systemic homeostasis, reflecting key features of aging. However, experimental evidence directly demonstrating accelerated aging in patients with FA is still lacking, and although no studies have examined the external phenotype of FA from the perspective of aging, its cellular and molecular defects, particularly in DNA repair, chromatin regulation, and mitochondrial function, recapitulate the mechanisms underlying accelerated aging.

FA shares similarities with syndromes such as Werner syndrome (WRN1 mutations), Hutchinson-Gilford progeria (LMNA mutations), and Cockayne syndrome (ERCC6/ERCC8 mutations) (Hennekam, 2020), all of which affect genome stability. FANC proteins have diverse roles, being ICL repair the most well-known, however, they might not be limited to this sole activity. Results from several studies appear promising, as they highlight new molecular mechanisms underlying FA disease progression and, simultaneously, alternative therapeutic pathways.

Taken together, FA provides a valuable model to study accelerated aging, illustrating the interplay between genomic instability, cellular stress responses, metabolic dysfunction, inflammation, and stem cell exhaustion. Studying FA offers unique insights into fundamental aging mechanisms and the relationship between aging and cancer, with significant implications for developing targeted therapies and understanding age-associated disease processes.
