# Supplementary material for: The tight bond between Fanconi anemia and aging
Source: Front Aging. 2026 Feb 24;7:1752160. doi: 10.3389/fragi.2026.1752160 (PMC12971690; doi:10.3389/fragi.2026.1752160)
Supplement: Supplementary file 1 [file Table1.docx]

Supplementary Material

# Supplementary Table

**Supplementary table 1. Fanconi anemia genes and their associated names**

| Gen | Name(s) | Locus |
| --- | --- | --- |
| *FANCA* | Fanconi Anemia complementation Group A | 16q24.3 |
| *FANCB* | Fanconi Anemia complementation Group B | Xp22.3 |
| *FANCC* | Fanconi Anemia complementation Group C | 9p22.3 |
| *FANCD1/BRCA2* | Fanconi Anemia complementation Group D1/ BReast CAncer gene 2 | 13q12.3 |
| *FANCD2* | Fanconi Anemia complementation Group D2 | 3p25.3 |
| *FANCE* | Fanconi Anemia complementation Group E | 6p21.3 |
| *FANCF* | Fanconi Anemia complementation Group F | 11p15 |
| *FANCG/XRCC9* | Fanconi Anemia complementation Group G/ X-Ray Repair, Complementing Defective, In Chinese Hamster, 9 | 9p13 |
| *FANCI* | Fanconi Anemia complementation Group I | 15q26.1 |
| *FANCJ/BRIP1/BACH1* | Fanconi Anemia complementation Group J/ BRCA1 interacting protein C-terminal helicase 1/ BTB Domain And CNC Homolog 1 | 17q22 |
| *FANCL/PHF9/FAAP43* | Fanconi Anemia complementation Group L/ PHD finger protein 9/ Fanconi anemia associated protein 43 | 2p16.1 |
| *FANCM* | Fanconi Anemia complementation Group M | 14q21.3 |
| *FANCN/PALB2* | Fanconi Anemia complementation Group N/ Partner And Localizer of BRCA2 | 16p12 |
| *FANCO/RAD51C* | Fanconi Anemia complementation Group O/ RAD51 paralog C | 17q25.1 |
| *FANCP/SLX4* | Fanconi Anemia complementation Group P/ SLX4 structure-specific endonuclease subunit | 16p13.3 |
| *FANCQ/XPF/ERCC4* | Fanconi Anemia complementation Group Q/ Xeroderma Pigmentosum, Complementation Group F/ Excision Repair Cross-Complementing Rodent Repair Deficiency, Complementation Group 4 | 16p13.12 |
| *FANCR/RAD51* | Fanconi Anemia complementation Group R/ RAD51 recombinase | 15q15 |
| *FANCS/BRCA1* | Fanconi Anemia complementation Group S/ BReast CAncer gene 1 | 17q21 |
| *FANCT/UBE2T* | Fanconi Anemia complementation Group M/ ubiquitin conjugating enzyme E2 T | 1q32.1 |
| *FANCU/XRCC2* | Fanconi Anemia complementation Group U/ X-ray repair cross complementing 2 | 7q36.1 |
| *FANCV/REV7/MAD2L2* | Fanconi Anemia complementation Group V/ REV7 Homolog/ Mitotic Arrest Deficient 2-Like Protein 2 | 1p36 |
| *FANCW/RFWD3* | Fanconi Anemia complementation Group W/ RING finger and WD repeat domain-containing protein 3 | 16q23.1 |
| *FANCX/FAAP100* | Fanconi Anemia complementation Group X/ Fanconi anemia associated protein 100 | 17q25.3 |
