# Supplementary material for: The tight bond between Fanconi anemia and aging
Source: Front Aging. 2026 Feb 24;7:1752160. doi: 10.3389/fragi.2026.1752160 (PMC12971690; doi:10.3389/fragi.2026.1752160)
Supplement: Supplementary file 2 [file Table2.docx]

Supplementary Material

# 2 Supplementary Table

**Supplementary table 2. Summary of experimental evidence supporting aging hallmarks in Fanconi anemia**

| Category | Hallmark | Species/Model | Findings | Ref. in the main manuscript |
| --- | --- | --- | --- | --- |
| Primary | Genomic instability | Human FA Peripheral blood | Crosslinking agents’ sensitivity | Auerbach, 2009; Schroeder et al., 1964 |
|  |  | Human and mouse/ *FANCA* or *FANCD2* deficient | R-loop accumulation | García-Rubio et al., 2015 |
|  | Telomere attrition | Human FA | Short telomeres | Hanson et al., 2001 |
|  |  | Human lymphocytes complementation group FA-D2 | Multiple types of telomeric abnormalities | Joksic et al., 2012 |
|  |  | Human lymphocytes complementation group FA-A, FA-G and FA-L | Telomere shortening | Shah et al., 2021 |
|  |  | *Fancc -/-* mice | Telomere attrition associated with high cell turnover | Rhee et al., 2010 |
|  | Epigenetic alterations | Human FA: peripheral blood mononuclear cells (PBMC) from blood samples | Decreased expression of epigenetic chromatin modification genes *DNMT1*, *DNMT3β* *CIITA, PAK1, RNF20, HDAC2, HDAC9, HDAC10 and HDAC11* | Belo et al., 2015 |
|  |  | Human fibroblasts *FANCC* -/- | Decreased H4K16 acetylation | Renaud et al., 2016 |
|  |  | Human cell line *FANCA* deficient | H4 hypoacetylation of replication fork-associated histones | García-de-Teresa et al., 2024 |
|  |  | Human cell line *FANCP/SLX4* deficient | Upregulated LINE-1 retro transposition | Brégnard et al., 2016 |
|  | Loss of proteostasis | *Fancd2-/-* mice | Increased protein synthesis | Kovuru et al., 2024 |
|  | Disabled macroautophagy | Mouse embryonic fibroblasts *Fancc* -/- | Virophagy and mitophagy disruption | Sumpter et al., 2016 |
|  |  | Mouse *Fancc -/-* infected with ZIKV | Increased infection and autophagy protein levels in various brain regions | Tiwari et al., 2020 |
|  |  | Human FA PBM from blood samples | Down expression of autophagy-associated genes | Zipporah et al., 2020 |
|  |  | HeLa *FANCL* -/- | Overexpression of Parkin (which regulates mitophagy) | Beesetti et al., 2022 |
| Antagonistic | Cellular senescence | Human melanoma cell lines + siFANCA/siFANCD2 | Increase of SA-β Gal, p53, p21 and p27, intracellular ROS accumulation | Bourseguin et al., 2016 |
|  |  | IMR90-hTERT y WI38-hTER cell lines + siFANCD2 | Increase in SAHF cells, p16, p21 and p53 | Helbling-Leclerc et al., 2019 |
|  | Mitochondrial dysfunction | Human fibroblasts *FANCD2* -/- | Increased ROS, altered mitochondrial morphology and decreased ATP levels | Kumari et al., 2014 |
|  |  | HCT116 *FANCD2 -/-* and HeLa *FANCD2 -/-* | FANCD2 participates in the mitochondrial unfolded protein response, the FANCD2/FANCI-UBL5 axis sense mitochondrial status. | Fernandes et al., 2021 |
|  | Deregulated nutrient-sensing | Human FA with oral administration of uniformly 13C-labeled glucose | Sustained hyperglycemia  Reduced energy expenditure  Persistent ketogenesis  Altered amino acid metabolism | Vicente-Muñoz et al., 2025 |
|  |  | Human FA monocytes | Increased gene expression of *TPH1* | Bartlett et al., 2021 |
|  |  | Human lymphoblasts complementation group FA-C | Reduction in tyrosine phosphorylation of the insulin receptor | Li et al., 2012 |
| Integrative | Stem cell exhaustion | Human FA HSPC | Overexpression of *p53* | Ceccaldi et al., 2012 |
|  |  | Human FA HSPC | Overexpression of *MYC* | Rodríguez, Zhang, et al., 2021a |
|  |  | *Fancd2-/-* mice  HSPC mouse  FA HSC human and mouse | Overexpression of *TGF beta* | Rodríguez et al., 2022; Rodríguez et al., 2021b ; Zhang et al., 2016 |
|  |  | Human FA MSC | Reduced proliferation ability *in vitro* | Mantelli et al., 2015 |
|  |  | DKO *Fancc/g* mouse *MSC* | Reduced proliferation ability and enhanced adipogenic differentiation | Zhou et al., 2017 |
|  | Altered intercellular communication | Human fibroblast complementation group FA-A | Decreased intracellular Ca^2+^ levels | Usai et al., 2015 |
|  | Chronic inflammation | *Fanca*-/-, *Fancc-/-* and *Fancg*-/- mice | Bone marrow failure driven by cytokine exposure | Li et al., 2004; Si et al., 2006 |
|  |  | Human lymphoblast complementation group FA-C | Overexpression of *IL-1RA*, *MIC-1* and *HSP-70* | Zanier et al., 2004 |
|  |  | Human lymphoblast complementation group FA-C | Time-dependent TNF- α overproduction | Briot et al., 2008 |
|  |  | Human fibroblast complementation group FA-D2 and    FA-C | Increased TNF- α and NF- κB activation | Matsushita et al., 2011 |
|  |  | Human lymphocytes complementation group FA-A | Increased IL-1ß | Ibáñez et al., 2009 |
